# Comment on: The Effect of Pedal Pump Lymphatic Technique Versus Passive Recovery Following Maximal Exercise: A Randomized Cross-Over Trial

**DOI:** 10.1186/s40798-022-00443-w

**Published:** 2022-04-11

**Authors:** Bruno Bordoni

**Affiliations:** Department of Cardiology, Institute of Hospitalization and Care, Foundation Don Carlo Gnocchi IRCCS, S Maria Nascente, Via Capecelatro 66, 20100 Milan, Italy

## To the Editor

I read with interest the article by DiFrancisco-Donoghue et al., a randomized cross-over trial carried out on healthy subjects and with the aim of reducing post-anaerobic metabolites (blood lactate accumulation), through an osteopathic manual approach for the stimulation of lymphatic circulation [1]. I would like to bring to your attention some clarifications.

In the text we can read that great importance is given to lactic acid; the latter is considered to be one of the most important causes for contractile fatigue of the skeletal muscle and for the onset of delayed muscle soreness (DOMS) [1]. During a muscular effort the demand by the contractile fibers (cross-bridge cycling and ionic pumping) of adenosine triphosphate (ATP) can increase by 100 times compared to a resting condition [2]. ATP is broken down several times, with an intracellular increase in inorganic phosphorus (Pi) which can reach about 500% more than at the beginning of the contraction [3]. The increase in Pi, in conjunction with the increase in acidity (although it is a minor cause), causes peripheral contractile fatigue. In particular, Pi binds to calcium, making calcium unable to bind to myosin, and causing the contraction to stop [4]. Furthermore, the Pi-bound calcium fails to return to the sarcoplasmic reticulum as it has a larger size, persisting in the sarcoplasm of the fiber; this event makes the intracellular environment "toxic" [5]. Finally, Pi is able to enter the sarcoplasmic reticulum and bind to calcium before the same calcium can enter the sarcoplasm; in this way, free calcium is reduced with a decrease in the number and strength of actomyosin bridges [5]. Pi is probably the most important metabolic cause of muscle breakdown in a regimen of intense anaerobic activity and not the increase in the formation of lactic acid.

Lactic acid postpones the onset of peripheral muscle fatigue. During muscle contraction in an intense anaerobic regime, there is an increase in the extracellular potassium concentration, in which increase induces a decrease in the excitability of the sarcolemma [6, 7]. Lactic acid, in particular hydrogen, accumulates outside the sarcolemma, protecting and trying to restore the correct membrane voltage, counteracting the inhibiting effect of extracellular potassium [7]. The relationship between the presence of DOMS and the production of lactic acid is not real: lactic acid does not cause DOMS [8].

Another reflection concerns the venous/lymphatic return. Osteopathy teaches that the diaphragm is an extraordinarily important area for bodily health [9]. During inhalation, abdominal pressure increases, and negative intrathoracic pressure increases, with an increase in the collapsibility of the inferior vena cava; these mechanisms facilitate venous flow to the cardiorespiratory system [10].

The diaphragm collects all the lymph from the viscera and abdominal muscles, from the lower limbs and in a small portion collects the parietal pleural lymph from the lower lung lobe [11]. The entire periphery of the diaphragm is rich in stomata with valves; the lymph is collected in lacunae and sent to the lymphatic vessels of the diaphragm, and finally, toward the Chyli cistern [11].

## Data Availability

Not applicable.
